# Mechanical Properties and Microstructure of Inconel 718 Lattice Structures Produced by Selective Laser Melting Process

**DOI:** 10.3390/ma17030622

**Published:** 2024-01-27

**Authors:** Sebastian-Marian Zaharia, Camil Lancea, Adam Kruk, Grzegorz Cempura, Adam Gruszczyński, Lucia-Antoneta Chicos, Mihai Alin Pop

**Affiliations:** 1 Department of Manufacturing Engineering, Transilvania University of Brasov, 500036 Brasov, Romania; zaharia_sebastian@unitbv.ro (S.-M.Z.); l.chicos@unitbv.ro (L.-A.C.); 2 Faculty of Metals Engineering and Industrial Computer Science, AGH University of Krakow, 30 Mickiewicza Ave, 30-059 Krakow, Poland; kruczek@agh.edu.pl (A.K.); cempura@agh.edu.pl (G.C.); gruszcz@agh.edu.pl (A.G.); 3 Department of Materials Science, Transilvania University of Brasov, 500036 Brasov, Romania; mihai.pop@unitbv.ro

**Keywords:** selective laser melting, lattice structures, microhardness, heat treatment, compressive behavior

## Abstract

This article presents the results of an analysis regarding the microstructure, mechanical strength, and microhardness of two kinds of samples built through selective laser melting with Inconel 718, the most frequently used alloy in metal additive manufacturing due to its excellent mechanical properties. The sample geometry was made up of two types of lattice structures with spherical and hyperbolical stiffness elements. The goals of these studies are to determine how homogenization heat treatment influences the microhardness and the mechanical properties of the specimens and to identify the structure with the best mechanical properties. The analysis showed that heat treatment was beneficial because the regular dendritic structure disappears, the δ phase precipitates at the grain boundaries, and both the γ and γ″ phases dissolve. It has also been shown that the structures with hyperbolical stiffness elements have better compressive strength than the structures with the elliptical structures, with a 47.6% increase for the as-fabricated structures and an approximate 50% increase for the heat-treated structure.

## 1. Introduction

Lattice structures were designed to be able to save material and to create lighter-than-traditional materials with characteristics close to their traditional counterparts [1,2,3]. These structures are characterized by stiffness, low weight, and high resistance to stress factors, especially mechanical stress [4,5]. The lattice structures arise from an attempt to mimic the high strength-to-mass ratios seen in nature, such as honeycomb design, bone structures, and insect wings, and, most notably, from their use as workpieces for aerospace industries; they have been used more recently for nuclear energy components, as well as in the petroleum and automotive industries [6,7].

Experience has demonstrated that the geometric shape of the lattice cell parts was insufficient to support the loads for which they were designed. Because of this, new materials were required, in addition to geometric creativity, in order to form these structures in a way that would closely resemble the mechanical or thermal characteristics of parts made from solid materials using conventional processes. From previous studies [8,9,10,11], the mechanical properties of lattice structures (Young’s modulus, Bulk modulus, and yield stress) are affected by the structure of the investigated material as well as the volume fraction.

The large-scale production of these kinds of materials has been possible since 1980, when additive manufacturing (AM), also known as 3D printing, appeared on the market [12]. Nowadays, additive manufacturing processes have proven very useful for manufacturing parts with lattice structures made of polymer materials (when using 3D printing machines) and metals or alloys (when using selective laser melting machines). Recent research results [13] proved the capability of the SLM (Selective Laser Melting) process to manufacture components with better mechanical performances than forged or cast materials when these components are used at ambient temperatures. The parts with complex geometric shapes can be manufactured with considerable potential using SLM, a powder bed fusion additive manufacturing process [14,15,16].

The most used materials for these kinds of structures are alloys of titanium, aluminium, copper, nickel, Cr-Ni, stainless steel, etc., as follows: Ti6Al4V, AlSi10Mg, Cu-15Ni-8Sn, 316L stainless steel, Inconel 718, etc. Currently, metal parts may be manufactured using four AM technologies: binder jetting, powder-bed-based fusion, sheet lamination, and direct energy deposition [17]. The above-presented materials can be produced using powder-bed-based fusion, a subsection of AM techniques that involves processing metal powders [18].

One of the most used materials applied in metal additive manufacturing techniques [19] is Inconel 718 alloy (NiCr19Fe19Nb5Mo3). An Ni-based superalloy has good tensile strength, creep resistance, high-temperature corrosion resistance, fatigue resistance at high temperatures, and good weldability [20,21,22].

In a recent study [23], porous Inconel 718 alloy structures, manufactured by the SLM process with tetrahedral and diamond configurations, were tested in compression. After the tests, it was shown that the diamond porous structure presented better compression performance compared to the tetrahedral porous structure due to the reinforcement effect of the horizontal beam in the tetrahedral structure. Through the SLM process, Wang et al. [24] designed, topologically optimized, and additively manufactured lattice structures from Inconel 718, with the following topologies: metal lattices, namely, BCC, BCCZ, and honeycomb; and three types of triply periodic minimal surface structures: gyroid, primitive, and I-WP. Tensile tests indicated a linear dependence between the tensile strength of BCC, BCCZ, gyroidian, primitive and I-WP specimens, and relative density.

Due to the high temperatures developed on small surfaces during manufacturing, the parts obtained through SLM technologies accumulate very high internal stresses. For this reason, when the manufacturing ends, an ageing treatment is required to precipitate strengthening phases fully, homogenize the material microstructure, and increase its mechanical performances [25]. Because the mechanical properties of selective laser melted (SLMed) Inconel 718 alloy can be changed by modifying its microstructure, the heat treatment parameters are critical and must be optimized according to the desired mechanical properties.

In the present study, systematic research was performed regarding the influence of the heat treatment on the microstructure, the compressive strength, and the microhardness of a new lattice structure’s topology (with spherical and hyperbolical stiffening elements) manufactured from SLMed Inconel 718 alloy.

## 2. Materials and Methods

### 2.1. The Samples Geometry Design

**Figure 1:**
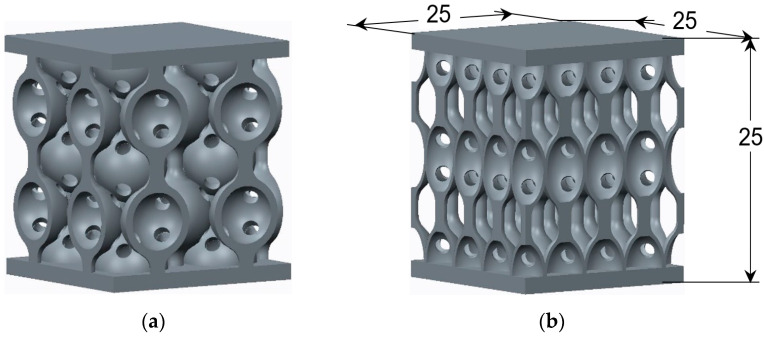
CAD Model of lattice structures: (**a**) with spherical stiffening elements; (**b**) with hyperbolical stiffening elements (dimensions in mm).

**Figure 2:**
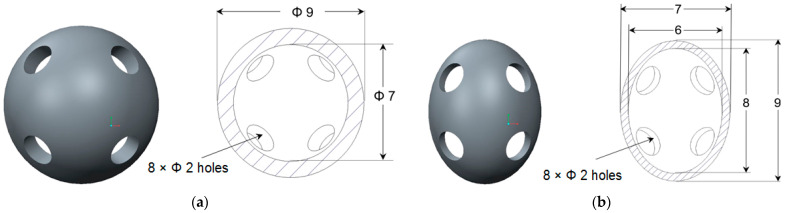
Dimensions CAD model of lattice structures: (**a**) with spherical stiffening elements; (**b**) with hyperbolical stiffening elements (dimensions in mm).

The analyzed samples have two types of lattice structures, having either spherical or hyperbolical stiffening elements. The sample dimensions are 25 × 25 × 25 mm (Figure 1), and the stiffening elements’ dimensions are indicated in Figure 2.

### 2.2. Inconel 718 Material

Inconel 718 is an austenitic nickel–chromium superalloy [26] well known for its stable mechanical properties, having high strength, high yield, excellent tensile, good oxidation and corrosion resistance, and favorable weldability at temperatures up to 700 °C [4,5,27]. Due to its outstanding behavior at high temperatures [27,28], Inconel 718 alloy has been extensively utilized for turbine blades, missile engines and nuclear reactors in the fields of aerospace, energy, and defence [29]. The Inconel 718 typical composition limits are 52 wt.% Ni; 19 wt.% Fe; 18 wt.% Cr; 5 wt.% (Nb + Ta); 3 wt.% MO; and traces of other elements [30].

As mentioned above, our samples are made of SLMed Inconel 718 alloy from gas-atomized Inconel 718 powder. The powder that was used as rough material [31], has a spherical shape with a diameter from 10 μm to 45 µm, a mass density of 8.2 g/cm^3^, and a thermal conductivity at 20 °C of 11.2 W/(m·K). The chemical composition is given in Table 1.

### 2.3. The SLM Process

The SLMed Inconel 718 alloy is manufactured layer by layer by melting a metal powder. The high internal stress that appears during the manufacturing process of Inconel 718 can cause cracking of the parts. High internal stress can be reduced by decreasing the cooling rate, heating the substrate plate, or subjecting the part to an appropriate heat treatment. At the same time, to increase the mechanical properties, the microstructure can be improved with a suitable heat treatment for eliminating defects and reducing internal stress [32,33,34].

**Figure 3:**
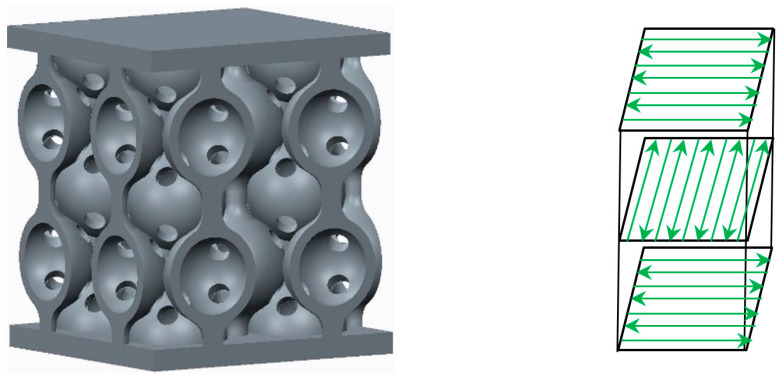
The cross-snake manufacturing strategy of the samples.

The SLM process was conducted using an SLM 280HL (SLM Solution Group AG, Lübeck Germany) system [35] equipped with 2 × 400 W yttrium fiber lasers and a 280 × 280 × 350 mm^3^ build chamber. Next, the main parameters of the SLM process were highlighted: the scanning speed—900 mm/s; the laser power—200 W; the hatch distance—120 μm; and the layer thickness—30 μm. The SLM manufacturing process was made in argon inert gas, and the gas consumption in the process was 2.5 L/min. The samples were vertically oriented for the building process, and a cross-snake hatch strategy was chosen (Figure 3).

Twenty Inconel 718 [31] samples were produced for this investigation: ten specimens with spherical stiffening elements and ten examples with hyperbolical stiffening elements. When the nominal dimension from the CAD file was compared with the measured lattice structures, the results (along the three axes) revealed a geometric deviation of about 0.03 mm.

### 2.4. The Heat Treatment of the Samples

**Figure 4:**
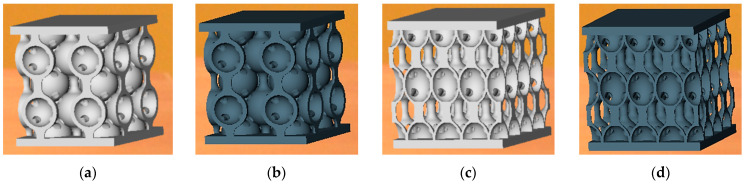
Samples having lattice structures with spherical stiffening elements before (**a**) and after the heat treatment (**b**); and samples having lattice structures with hyperbolical stiffening elements before (**c**) and after the heat treatment (**d**).

After the manufacturing process, the Laves phase that causes the segregation of Niobium decreases Inconel mechanical properties by preventing the strengthening phases γ′ and γ″. To improve its properties, a thermal treatment is needed to dissolve the Laves phase (when the subgrain boundaries disappear too), to release Niobium, and to precipitate γ′/γ″ phases. Therefore, to obtain a homogeneous microstructure with good mechanical properties, the heat treatment temperature should reach 1080 °C [13,25,36]. The lattice specimens were subjected to a homogenization heat treatment (Figure 4), as presented in Table 2.

### 2.5. Microstructure Analysis

The TEM analysis was performed using the 200 kV Transmission Electron Microscope Tecnai G2 20 TWIN (TEM, FEI, Hillsboro, OR, USA).

### 2.6. Microhardness Tests

**Figure 5:**
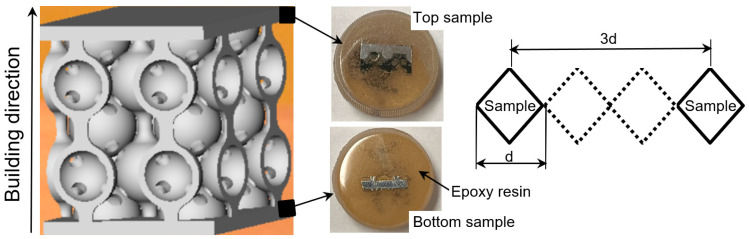
Schematic representation of the location where the specimen’s microhardness was determined.

To assess the microhardness of the designed structures, the samples were sliced in a vertical cross section parallel to the building direction (Figure 5). Then, using the grinding equipment (Phoenix Beta, Buehler, IL, USA), cut samples were embedded in epoxy resin and ground with sandpaper while gradually varying the granularity (600, 1200, 1500, 2000, and 2500). Ten microhardness measurements were made for each sample using the Future-Tech FM-700 microhardness tester (Future-Tech Corp., Tokyo, Japan) at a load of 100 gf. and a loading duration of 15 s. For the Vickers test, the distance between indents must be at least three times the d length (Figure 5).

### 2.7. Compression Tests

**Figure 6:**
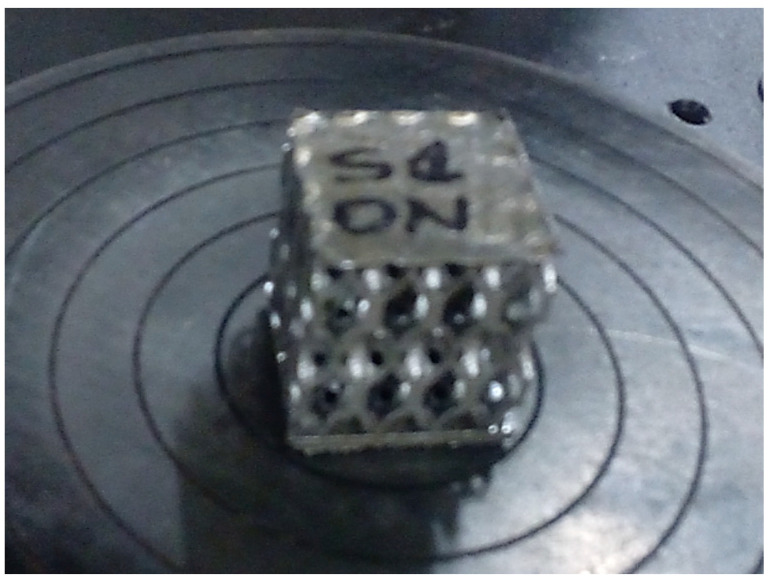
Compression testing of the sample with hyperbolical stiffness.

Compression testing was performed on 20 lattice structures of Inconel 718 having spherical (10 samples) or hyperbolical (10 samples) stiffening elements, manufactured by SLM (Figure 6). To determine the compression strength, the ends of the samples and fixture-bearing blocks were cleaned with acetone, placed in the manufacturing position, and subjected to static flatwise compression.

In good agreement with ASTM standards [41], the compression tests were conducted on a WDW-150S testing machine (Jinan Testing Equipment IE Corporation, Jinan, China) with a crosshead speed of 2 mm/min. The specimens were carefully aligned on the machine to ensure a uniform loading. A displacement transducer with a 0.01 mm precision was installed on the drive screw of the mechanical test device.

To determine the compression performance of the lattice structures, the dimensions of the specimens (25 mm × 25 mm × 25 mm) were introduced into the MaxTest V1 software system of the testing equipment. Using the relations (1) and (2), specific to compression tests [42], the compressive strength (σ_c_) and compressive modulus (E_c_) were determined as follows:

(1) $$\sigma_{c}=\frac{P_{c}}{A_{c}},$$

(2) $$E_{c}=\frac{m\cdot t}{A_{c}},$$

where P_C_ is the ultimate load on the compression tests (N); A_C_ is the cross sectional area of the lattice specimens (mm^2^); m is the slope of the tangent to the initial straight-line portion of the load–deflection curve (N/mm); and t is the nominal facing thickness (mm).

The equipment’s software was programmed by the manufacturer with the specified calculation equations for mechanical tests, and the test report automatically generated the modulus compression and compression strength values.

## 3. Results and Discussion

For evaluating the influence of homogenization heat treatment on the microstructure, the compressive strength, and the microhardness of two different lattice structures manufactured with SLMed Inconel 718 alloy, the tests and analyses presented below were made.

### 3.1. Microstructure Observation and Analysis

**Figure 7:**
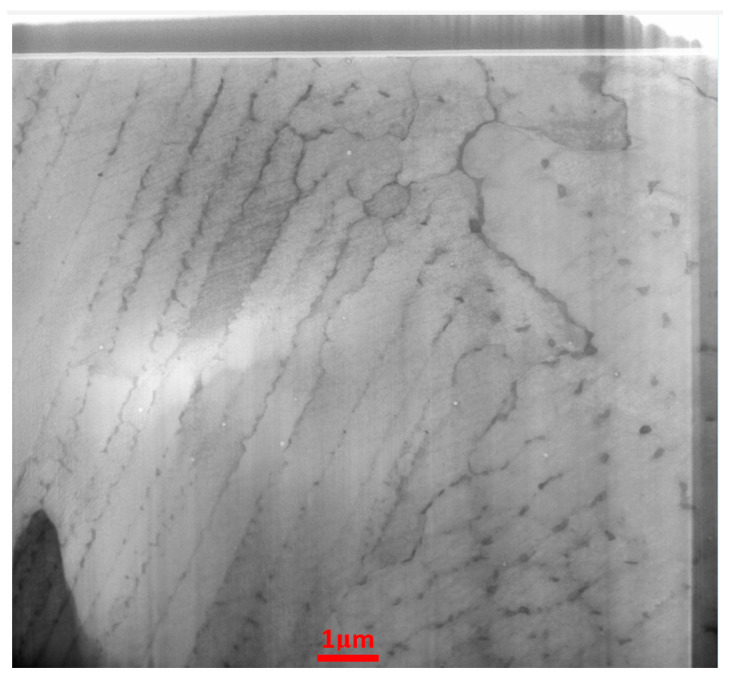
Lamella for TEM examination.

As mentioned before, two sets of heat-treated and untreated samples were subjected to microstructure analysis. Using the electron microscope Zeiss Neon 40 EsB Crossbeam SEM-FIB (Carl Zeiss, Oberkochen, Germany), the focused ion beam (FIB) technique was applied to prepare the lamellae for TEM examinations (Figure 7). The samples were extracted from the as-built and heat-treated samples’ cross sections.

**Figure 8:**
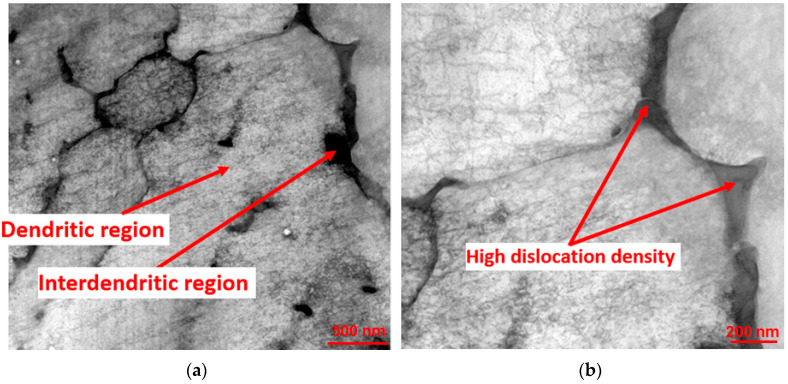

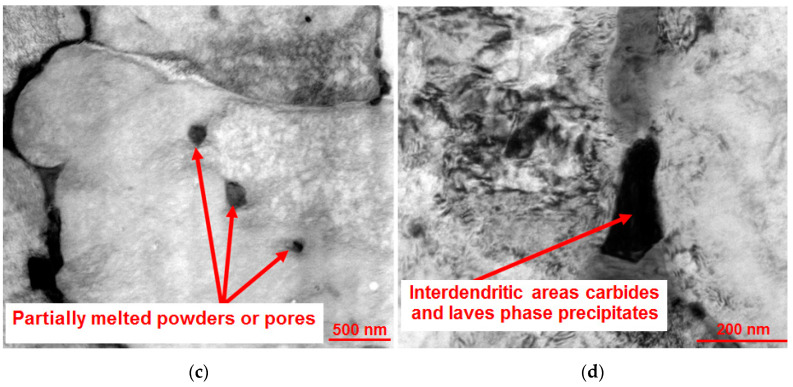
The sample before heat treatment: (**a**) dendrites and interdendritic gaps; (**b**) high dislocation density and thermal cracks; (**c**) zones with partially melted powders and/or pores; (**d**) zone with interdendritic areas carbides and laves phase precipitates.

**Figure 9:**
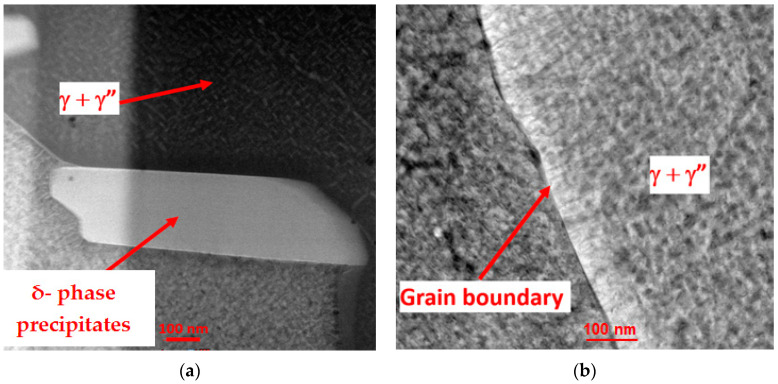
The sample after heat treatment: (**a**) δ phase precipitates at the grain boundaries; (**b**) γ and γ″ phases precipitates in the γ matrix.

Each set was composed of 10 samples. The homogenization heat treatment of Inconel 718 samples significantly affected the overall microstructural characteristics, as shown in Figure 8 and Figure 9. It is relevant that the as-built SLMed Inconel 718 samples had average surface roughness values ranging from 19 to 24 μm [43].

Dendrites, interdendritic gaps (Figure 8a), a high dislocation density, and thermal cracks (Figure 8b) all occurred due to the high scan speed and repeated rapid melting and solidification processes. Dendrite morphology also lowered tensile strength [44,45]. During the building process, partially melted powders and/or pores (Figure 8c) on the outer surface of the metallic parts were observed in the microstructure of components manufactured by selective laser melting [43]. Our previous research on Inconel 718 [46,47,48] revealed that this type of image comes from the interdendritic areas. Therefore, the Laves phase, as well as eutectic carbides, should be present. Also, based on our previous research [46,47,48], it can be stated that the marked phase (taking size into account) can be a carbide. The particles visible above and below (with grey contrast) were most likely in the Laves phase.

After the homogenization heat treatment, the regular dendritic structure disappeared (Figure 9). In general, after heat treatment, this alloy precipitates a delta phase with the morphology of the particle. In the background, delta-phase precipitates were observed (Figure 9a). Because the presence of the delta phase has been widely reported by previous authors [49], no detailed investigations were carried out. In addition, we also observed the γ and γ″ phases precipitating in the γ matrix (Figure 9b).

### 3.2. Microhardness Tests

Microhardness testing of Inconel 718 samples produced using SLM technology is a heavily investigated topic. In this regard, depending on the sample geometry, some research [50,51] has found insignificant microhardness fluctuation along the construction height, but others [52,53] have found that microhardness decreases with build height.

In the present case, the test findings revealed a noticeable difference in microhardness values on the vertical cross-section plane surface on the bottom and top sections and for as-fabricated and heat-treated specimens.

The mean microhardness values in the as-fabricated spherical samples were 342.1 HV (34.6 HRC) on the top section and 379.4 HV (38.3 HRC) on the bottom section, with a 10.1% increase from top to bottom (Table 3).

With a 9.2% increase from top to bottom, the average microhardness value in the heat-treated spherical samples was 446.2 HV (44.8 HRC) on the top of the sample and 487.2 HV (48.1 HRC) on the bottom. The mean microhardness values in the as-fabricated elliptical samples were 299.1 HV (29.6 HRC) on the top section and 326.1 HV (32.8 HRC) on the bottom section, with a 9.04% increase from top to bottom. With a 4.35% increase from top to bottom, the average microhardness value in the heat-treated elliptical samples was 440.5 HV (44.3 HRC) on the top of the sample and 459.7 HV (46.9 HRC) on the bottom.

The aforementioned results showed that microhardness reduces with build height even for specimens that have been heat treated. The heat-treated specimens showed an increase in microhardness of 30.4% for the spherical specimens (47.3% for the elliptical specimens) investigated in the upper section and 28.43% for the spherical specimens (40.9% for the elliptical specimens) investigated in the lower section, when compared to the as-fabricated specimens.

These results are justified because the bottom area of the sample structure suffered repeated heating cycles during the SLM process, which was assimilated to a heat treatment. This process led to enhanced precipitation hardening and was responsible for the higher microhardness at the bottom of the sample [54]. The lower microhardness value at the top of the lattice structure could be attributed to a decrease in the strengthening phase. The laser only passes over the surface of the sample structure once, and no subsequent heat treatments are performed. Other studies [19,55,56] found similar results on microhardness changes.

Specific to the SLM process, it constitutes a lower porosity at the bottom, which means a lower density and therefore a higher microhardness [57]. In a recent study [55], it was shown that more γ″ was found at the bottom of an as-fabricated specimens, which explains the decrease in microhardness they observed with height. The cause of this was speculated to be due to growth and precipitation of γ″ in subsequent hatches and layers as allowed by Nb segregation [55]. Seede et al. [58] reported a difference in microhardness of about 11% between the upper area (260 HV) and the bottom area (289.1 HV) of the Inconel 718 part after the homogenization process. This is explained by the existence of thin columnar grains left over by the SLM process at the bottom of the Inconel 718 homogenized parts [58].

### 3.3. Compression Tests

To determine the best lattice structure, two different types of samples were obtained through AM, and different lattice structures were subjected to compression tests to determine which ones had better strength under compression.

Two sets of samples were built, each consisting of ten samples made from the identical lattice structure. Four sets of five samples each were used for the compression tests, which are as follows: five as-fabricated lattice structures with spherical stiffening shapes (As-SSS); five as-fabricated lattice structures with hyperbolical stiffening shapes (As-HSS); five heat-treated lattice structures with spherical stiffening shapes (HT-SSS); five heat-treated lattice structures with hyperbolical stiffening shapes (HT-HSS).

**Figure 10:**
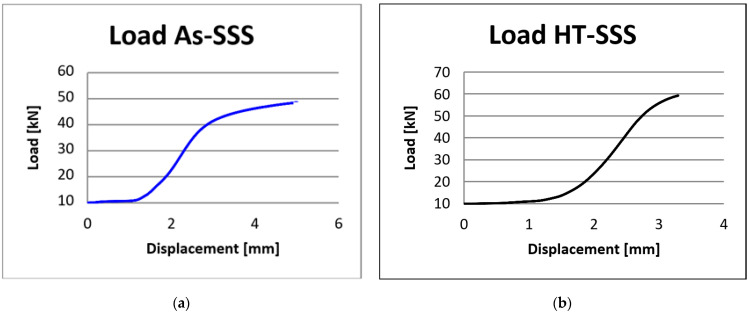
Load–displacement curves: (**a**) as-fabricated lattice structures with spherical stiffening shapes (As-SSS); (**b**) heat-treated lattice structures with spherical stiffening shapes (HT-SSS).

**Figure 11:**
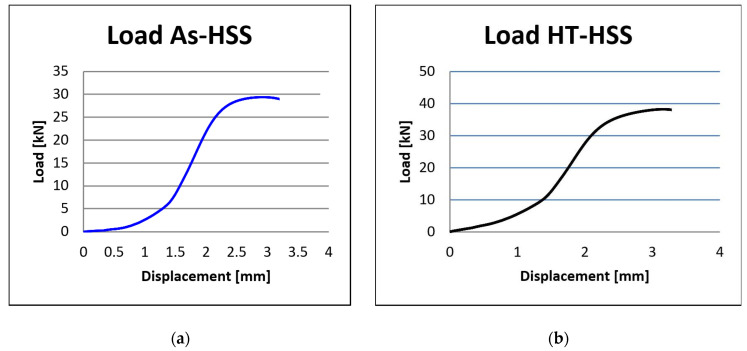
Load–displacement curves: (**a**) as-fabricated lattice structures with hyperbolical stiffening shapes (As-HSS); (**b**) heat-treated lattice structures with hyperbolical stiffening shapes (HT-HSS).

In this regard, twenty samples with a lattice structure were compressed until the fracture appeared. The load–displacement curves (Figure 10 and Figure 11) were computed by the WDW-150S universal testing machine’s software.

The values obtained from mechanical testing each group of five samples were averaged to construct the compression curve of load displacement, presented in Figure 10 for samples with spherical stiffening shapes, and in Figure 11 for samples with hyperbolical stiffening shapes.

When evaluating the as-fabricated AS-SSS, the test machine’s plate was close but not in contact with it. However, this gap in the load–displacement curve does not affect the compression test result.

Consequently, there was a distance at which the testing device just registered displacement in the absence of specimen loading. The load–displacement behavior of the twenty samples under static flatwise compression is shown in Figure 10 and Figure 11. The load–displacement curves presented three different regions:

- the distance between the sample and the machine plate causes displacement to rise while the applied force stays constant. Within this phase, the plate approaches the sample without making direct contact.
- the linear dependence zone occurs when the force applied to the sample grows until it reaches the plateau zone.
- the high point area where the part fails owing to an increase in force.

**Figure 12:**
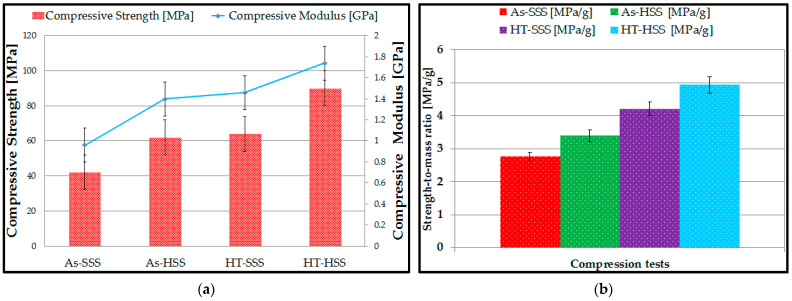
Compressive test results: (**a**) mean values of compressive strength; (**b**) analysis of the strength-to-mass ratio of lattice structures.

The compression testing device’s software enabled the computation of mechanical characteristics, including compressive strength and compressive, modulus for the lattice structures. As shown in Figure 12a, the compressive modulus fluctuated between 0.96 GPa for As-SSS specimens (with a standard deviation of ±0.01 Gpa) and 1.74 Gpa for HT-HSS specimens (with a standard deviation of ±0.02 Gpa), whereas the compressive strength ranged from 42 Mpa for As-SSS specimens (with a standard deviation of ±3.2 Mpa) to 90 Mpa for HT-HSS specimens (with a standard deviation of ±4.1 Mpa).

Furthermore, as can be seen in Figure 12a, the lattice structures with hyperbolical stiffening shapes—both as-fabricated and heat-treated—performed better in compression tests than the lattice structures with spherical stiffening shapes, demonstrating higher compressive strengths and compressive modulus. The compressive performance of the lattice structures was analyzed using the specific strength-to-mass ratio (Figure 12b).

Following the mechanical tests, the phenomenon of local buckling [59] of the cells appeared, and the specimens with a smaller curvature (hyperbolical stiffening shapes) showed a higher compressive strength compared to specimens with a large curvature (lattice structures with spherical stiffening).

Since mass is a determining factor in the analysis of the mechanical performance of Inconel 718 lattice structures, in this study, the specific strength-to-mass ratio was determined, and the following outcomes were drawn:

- Based on the compression flatwise tests (for as-fabricated), the lattice structures with hyperbolical stiffening shapes presented the best performances (23% higher compared to lattice structures with spherical stiffening shapes);
- Based on the compression flatwise tests (for heat-treated), lattice structures with hyperbolical stiffening shapes presented the best performances (17.3% higher compared to lattice structures with spherical stiffening shapes).

## 4. Conclusions

For the first time, two different types of lattice structures containing spherical and hyperbolical stiffening elements were produced directly by the laser-selective melting technique in this study. The limitations of the selective laser melting technology were considered during the design and manufacturing of the two topologies. Thus, the core was perforated to remove the metallic powders from the stiffening cells inside. After being built directly using SLM technology, the two kinds of parts were subjected to a homogenization heat treatment to enhance their mechanical properties.

TEM microscopy techniques were employed to analyze the microstructure of as-fabricated and heat-treated materials, and compression and microhardness tests were used to determine their mechanical properties. The Inconel 718 lattice samples revealed a common microstructure of SLM-manufactured samples in the TEM investigations. The microscopic images were studied before and after the homogenization heat treatment, and the results demonstrate the heat treatment’s benefits.

The results of the compression tests also revealed the following: after the homogenization heat treatment, the two lattice structures showed an increase of 45.1% (lattice structures with hyperbolical stiffness elements) and 52.3% (lattice structures with spherical stiffness elements) of the compressive strength. As-fabricated lattice structures with hyperbolical stiffness elements showed a 47.6%-higher compressive strength compared to as-fabricated lattice structures with spherical stiffness elements; heat-treated lattice structures with hyperbolical stiffness elements had a 40.6%-higher compressive strength than heat-treated lattice structures with spherical stiffness elements.

On a vertical cross section of the as-fabricated and heat-treated samples, the microhardness tests were conducted. The microhardness of the heat-treated structures with elliptical stiffness elements increased by approximately 29%, and the microhardness of the lattice structures with elliptical stiffness elements increased by approximately 44% compared to the non-heat-treated specimens. These findings were reached after the microhardness tests. Microhardness decreases with the build height of the as-fabricated and heat-treated lattice structures.

The novelty of this study includes the demonstration of the metal additive manufacturing of innovative lattice structures with spherical and hyperbolical stiffening elements, for which the mechanical performances (compression and microhardness) were determined before and after the homogenization heat treatment. The proposed lattice samples’ low mass and high mechanical properties make them highly valuable for applications in materials engineering, bioengineering, aerospace, automotive, and military industries.

## Figures and Tables

**Table 1:** Inconel 718 powder: chemical composition (nominal) wt.% [31].

Element	IN718
Ni	50.00–55.00
Cr	17.00–21.00
Fe	Bal
Ta + Nb	4.75–5.50
Mo	2.80–3.30
Ti	0.65–1.15
Al	0.20–0.80
Cu	0.30
C	0.08
Si, Mn	0.35 each
B	0.006
Co	1.00
P, S	0.015 each

**Table 2:** Homogenization solution aging thermal treatment [37,38,39,40].

Standard	Treatment Type	Temperature	Holding Time	Cooling
AMS 5664	Homogenization solution aging	1080 °C	1.5 h	Air cooling
		980 °C	1 h	Air cooling
		720 °C	8 h	Furnace cooling at 55 °C/h to 620 °C
		620 °C	8 h	AC

**Table 3:** Microhardness values on the vertical cross-section plane.

Sample Type	Section Position	Medium (HV)	Medium (HRC)
Spherical as-fabricated	top	342.1	34.6
Spherical as-fabricated	bottom	379.4	38.3
Spherical heat-treated	top	446.2	44.8
Spherical heat-treated	bottom	487.2	48.1
Elliptical as-fabricated	top	299.1	29.6
Elliptical as-fabricated	bottom	326.1	32.8
Elliptical heat-treated	top	440.5	44.3
Elliptical heat-treated	bottom	459.7	46.9

## Data Availability

Data are contained within the article.
